# Preparation of Cellulose-Based Activated Carbon Fibers with Improved Yield and Their Methylene Chloride Adsorption Evaluation

**DOI:** 10.3390/molecules28196997

**Published:** 2023-10-09

**Authors:** Jin-Soo Jeong, Byung-Joo Kim

**Affiliations:** 1 Materials Application Research Institute, Jeonju University, Jeonju 55069, Republic of Korea; 2 Department of Carbon Convergence, Composite Materials Engineering, Chonbuk National University, Jeonju 54896, Republic of Korea; 3 Department of Advanced Materials, Chemical Engineering, Jeonju University, Jeonju 55069, Republic of Korea

**Keywords:** cellulose, ammonium phosphate, activated carbon fiber, high yield, methylene chloride

## Abstract

The recent rapid growth of the battery industry has led to a rapid increase in methylene chloride emissions. Methylene chloride causes health and social problems in humans. In this study, cellulose-based activated carbon fibers (CACFs) with improved yield were prepared for the removal of methylene chloride. The concentration of ammonium phosphate in the pretreatment controlled the crosslink density of cellulose fibers and improved the yield. From the results, the specific surface area and total pore volume of cellulose-based activated carbon fibers pretreated with ammonium phosphate (AP-CACFs) were determined to be 1920–2060 m^2^/g and 0.83–1.02 cm^3^/g, respectively, and the total yield improved by 6.78–11.59% compared to that of CACFs (4.97%). In particular, a correlation between the textural properties of CACFs and methylene chloride adsorption/desorption behavior was obtained. This correlation can be used to develop efficient adsorbents for methylene chloride removal.

## 1. Introduction

Methylene chloride (MC) is a volatile organic compound used in vapor degreasing, metal cleaning, refrigerant chemical production, sealants, and adhesive removers (including adhesives, degreasers, and automotive products) [1]. It has harmful effects on the human body and the environment and may cause headaches, vomiting, difficulty breathing, air pollution, and destruction of the ozone layer. Recently, the electric vehicle market has grown rapidly, and the amount of MC emitted by the battery industry is inevitably increasing. Concerns have been raised about the impact of MC emissions on human health, and the need for its removal is increasing owing to the strengthening of environmental regulations [2,3,4].

The removal of harmful gases using porous adsorbents has been proposed as an innovative treatment technology [5]. Porous carbon materials are competitive adsorbents because of their large specific surface areas and tailored porous structures [6]. Activated carbon (AC) is mainly used as an adsorbent for volatile organic compounds. However, MC has a low boiling point (39.6 °C) and is emitted as a high-concentration vapor similar to the original solution. Therefore, to prevent the emission of high concentrations of MC in the workplace, it must be mixed with air at a high ratio. Thus, an AC with large pressure loss is disadvantageous for practical applications.

MC treatment technologies include reburning [7] and recovery [8]. The reburning process can generate highly corrosive substances, which consequently increase equipment maintenance costs. The recovery process includes filtration, adsorption, and absorption. Adsorption is a contaminant removal method using a porous adsorbent. Adsorption is relatively easy to manage and offers advantages of adsorbent reuse and resource recovery.

Adsorption mainly occurs in the micropores of porous substrates. Among the various porous carbon materials, activated carbon fibers (ACFs) have micropores that are mainly developed on the surface, whereas AC has micropores that are mainly developed inside. Therefore, ACF exhibits excellent adsorption performance with a shorter contact time than that of AC. Further, AC absorbs contaminants inside, and a considerable amount of time is needed to remove the adsorbed contaminants compared with that required for ACF [9,10,11,12].

Porous carbon materials are prepared from various precursors, such as cellulose and pitch [13,14,15]. Cellulose, which is the main component of plants and is almost inexhaustible, is produced at approximately 10^11^–10^12^ tons per year [16]. In addition, it is conducive to the development of micropores because of its loose molecular structure. Therefore, cellulose is one of the suitable precursors for MC adsorbents released in mixtures with air at a high ratio.

ACF can be prepared via physical or chemical activation. The chemical activation method [17] involves pretreatment of the precursor with oxidizing chemicals, such as phosphoric acid and potassium hydroxide, followed by pore formation in an inert atmosphere. In contrast, physical activation [18] involves carbonizing the precursor in an inert atmosphere and subsequently creating pores using carbon dioxide and steam. Chemical activation results in superior pore properties compared to those with physical activation. However, chemical activation can lead to issues such as device corrosion and secondary environmental pollution caused by the use of oxidizing agents. Therefore, physical activation offers significant advantages [19]. ACF is prepared through heat treatment such as stabilization, carbonization, and activation processes. The stabilization process is performed at about 200–300 °C, and the thermal stability is improved by oxidizing the precursor and crosslinking the molecular chain. The carbonization process involves heat treatment at about 800–1000 °C in an inert atmosphere (such as nitrogen and argon) and removes noncarbon atoms, such as nitrogen and oxygen. The thermal stability of cellulose is low due to its relatively low thermal decomposition temperature (approximately 300 °C) and the presence of a hydroxyl group (or oxygen); therefore, cellulose-based activated carbon fiber (CACF) has a low yield [20].

Studies have been conducted on the effects of various pretreatments on cellulose to improve its yield. Hagman et al. reported the dependence of cellulose on NaOH treatment (temperature and concentration) [21]. Jia et al. studied the phosphoric acid treatment of cellulose and reported its crystal structure [22]. Jocić et al. pretreated cellulose precursors with diammonium hydrogen phosphate (DAPH) and studied dimethoate removal from water [23]. Previous studies have mainly focused on the effects of various pretreatments on cellulose. Tsai et al. studied the adsorption of MC onto activated carbon [24]. However, little research has been conducted on the adsorption and behavior of activated carbon fibers and methylene chloride. Further, the adsorption of methylene chloride by activated carbon fibers remains unclear.

In this study, CACF was prepared for the removal of MC by pretreating a rayon-based cellulose precursor with ammonium phosphate. Ammonium phosphate improves the yield of cellulose during heat treatment because of its excellent thermal stability. Ammonium phosphates release phosphoric compounds and amino radicals during thermal decomposition, eventually enhancing the nitrogen content of the produced activated carbon. In other words, ammonium phosphate increases the crosslinking density of the cellulose precursor and facilitates the formation of graphite structures during carbonization. This, in turn, enhances the yield [25]. CACFs pretreated with ammonium phosphate (AP-CACFs) were evaluated for MC activity (MA), retentivity (MR), and working capacity (MWC). In particular, a correlation between the textural properties and the adsorption/desorption behavior of MC was obtained. This correlation is a potential factor in developing efficient adsorbents for MC.

## 2. Result and Discussion

**Figure 1:**
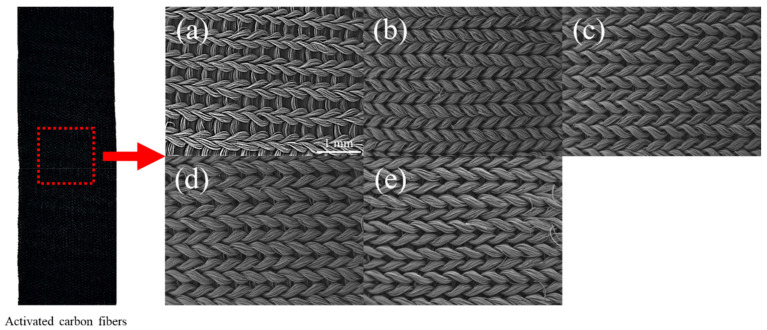
Scanning electron microscope images of cellulose-based activated carbon fiber (CACF) and ammonium phosphate pretreated cellulose-based activated carbon fiber (AP-CACF): (**a**) CACF, (**b**) AP025-CACF, (**c**) AP050-CACF, (**d**) AP075-CACF, (**e**) AP100-CACF.

The surface morphologies of nontreated cellulose-based activated carbon fiber (CACF) and cellulose-based activated carbon fiber pretreated with ammonium phosphate (AP-CACF) were analyzed using a scanning electron microscope (SEM), and the images are exhibited in Figure 1. As shown in Figure 1, the CACF and AP-CACF maintained their knitted shapes. In addition, the fiber spacing in the CACF was wider than that of the AP-CACF (Figure 1a). Generally, cellulose shrinks owing to heat, and the shrinkage caused by the same heat treatment is similar. Ammonium phosphate is known to play a role in crosslinking cellulose molecules, which may be due to the development of the crystal structure of cellulose during heat treatment (stabilization and carbonization) [26]. Therefore, AP-CACF shrinks more than CACF.

**Figure 2:**
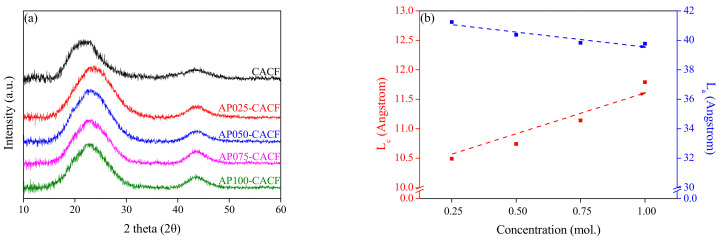
X-ray diffraction patterns (**a**) and structural characteristics (**b**) of cellulose-based activated carbon fibers pretreated with various concentrations of ammonium phosphate.

X-ray diffraction analysis is a useful method for analyzing the crystal structure of carbon materials. Figure 2 shows the X-ray patterns and grain sizes (L_c_ and L_a_) of the CACF and AP-CACF. In Figure 2a, the 002 peaks of CACF and AP-CACF were observed at approximately 22.07° and 23.60–24.24°, respectively. The 002 peak of typical graphite is observed at approximately 26.60°. The development of a graphite structure occurs as the 002 peak approaches 26.60°. Therefore, the crystal structure of AP-CACF is more developed than that of CACF. This is because ammonium phosphate increases the crosslinking density of cellulose, which due to the development of the crystal structure of AP-CACF occurs during the same heat-treatment process. In addition, in Bragg’s law, the interplanar distance is inversely proportional to 2θ, and thus, an increase in 2θ results in a decrease in the interplanar distance. This is consistent with the result that the AP-CACF contracted more than the CACF, as shown in the SEM image in Figure 1.

As exhibited in Figure 2b, the crystallite sizes (L_c_ and L_a_) of the CACF and AP-CACF were calculated using the Scherrer equation, where L_c_ and L_a_ are the stacking height and length, respectively [27]. In CACF, L_c,_ and L_a_ were 12.36 Å and 32.26 Å, respectively, whereas in AP-CACF, as the concentration of ammonium phosphate increased, L_c_ increased to 10.49–11.79 Å, and L_a_ slightly decreased to 41.24–39.77 Å. Therefore, as the concentration of ammonium phosphate increased, the crosslinking density and L_C_ increased. In addition, the crystallite sizes of CACF and AP-CACF were different. This may be due to the accelerated structural development of cellulose caused by ammonium phosphate. Based on the results, L_a_ decreased as the concentration of ammonium phosphate increased. As the ammonium phosphate concentration increased, the crosslinking density of the cellulose increased, and consequently, the crystallite size of the carbonized fibers increased. Therefore, the amorphous content decreased as the crystallite size increased, oxidizing more crystallite. Thus, during the same activation process, more crystallites were oxidized in AP025-CACF than AP100-CACF, and L_a_ slightly decreased accordingly.

**Figure 3:**
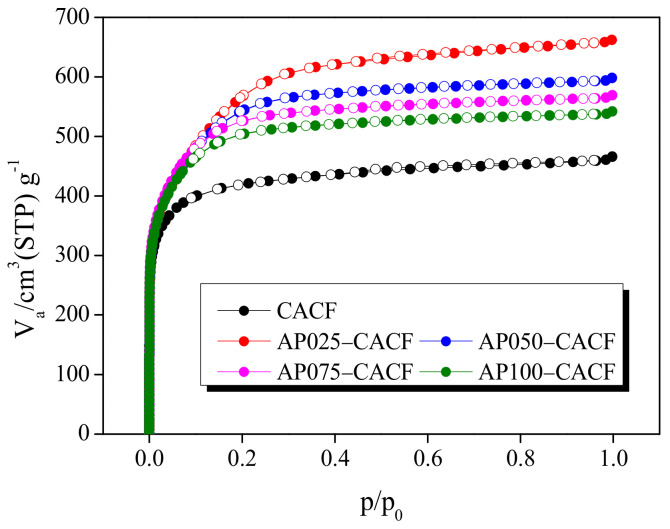
N_2_/77 K adsorption/desorption isotherm curves of cellulose-based activated carbon fibers pretreated with various concentrations of ammonium phosphate.

The isothermal adsorption/desorption curve (BET) is a useful method for analyzing the textural properties of porous carbon materials. Figure 3 exhibits the N_2_/77 K isothermal adsorption/desorption curves of CACF and AP-CACF. The isothermal adsorption/desorption curves of CACF and AP-CACF correspond to Type I of the IUPAC classification [28], and adsorption was mainly observed when the relative pressure (P/P_0_) was <0.2. Therefore, AP-CACF exhibited similar microporous structures [29]. These results indicate that micropores mainly developed because of a single-layer adsorption caused by the strong interaction between the pore wall of the activated carbon fiber (ACF) and the adsorbate (N_2_) [30]. In addition, hysteresis was hardly observed in the AP-CACF, which was determined to have wedge-shaped pores. However, weak hysteresis was observed in the CACF, which may be caused by the oxidation of the crystallite edges due to excessive activation. Therefore, CACF developed pot-shaped pores owing to oxidation at the crystallite edges.

Table 1 shows the textural properties of the CACF and AP-CACF. The specific surface area of AP-CACF decreased from 2060 to 1920 m^2^/g as the concentration of ammonium phosphate increased. Accordingly, the total pore volume of AP-CACF decreased to 1.02–0.83 cm^3^/g, and the micropore and mesopore volumes decreased to 0.96–0.80 cm^3^/g and 0.06–0.03 cm^3^/g, respectively. Generally, physical activation using steam is caused by the formation of micropores by first oxidizing the amorphous and then the crystallite edges to form mesopores [31]. In Figure 2b, as the ammonium phosphate concentration increased, L_C_ was increased by 10.49–11.79 Å, and the amorphous content was decreased. Therefore, the decrease in amorphous content, which influences the micropores, reduced the specific surface area and pore volume [32]. This result is consistent with the increase in the activation yield. As the concentration of ammonium phosphate increased, the fraction of micropores increased from 91.67% to 96.42%, whereas that of mesopores decreased from 8.33% to 3.58%.

The specific surface area and activation yield of CACF were 1570 m^2^/g and 25.65%, respectively, while the specific surface area and activation yield of AP050-CACF were 2030 m^2^/g and 29.22%, respectively. [33]. The specific surface area of AP050-CACF was 2060 m^2^/g, which was higher than that of CACF (1570 m^2^/g). However, the yield of AP050-CACF was 29.22%, which was higher than that of CACF (25.65%). This is because the pore structure could not develop further and collapsed owing to excessive oxidation during the activation process [33]. This is consistent with the hysteresis observed in the CACF exhibited in Figure 3.

**Figure 4:**
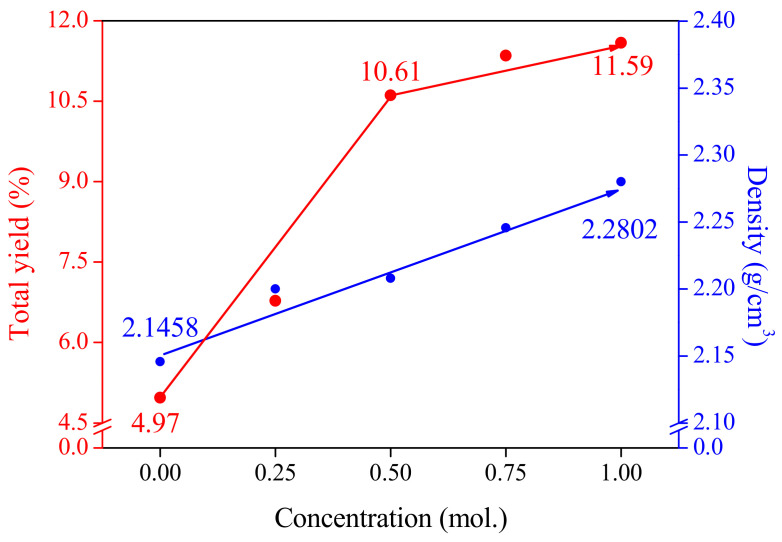
Total yield and density of cellulose-based activated carbon fibers pretreated with various concentrations of ammonium phosphate.

The total yields and densities of the CACF and AP-CACF are exhibited in Figure 4. The total yield represents the ratio of ACFs to the precursor and increased from 4.97% to 11.59% as the concentration of ammonium phosphate increased. However, the rate of yield increase was reduced in AP050-CACF. In contrast, the densities of the CACF and AP-CACF increased continuously as the concentration of ammonium phosphate increased. This may be due to the increase in cellulose crosslinking density caused by the ammonium phosphate pretreatment, which is consistent with the increase in L_c_ observed in Figure 2b. Consequently, ammonium phosphate increases the crosslinking density and is considered to improve the crystallite size of the carbonized fibers. Therefore, AP-CACF can have a better yield than CACF in the same activation process. This result is similar to the activation yield of AP-CACF in Table 1, which increased from 19.12% to 37.98%.

**Figure 5:**
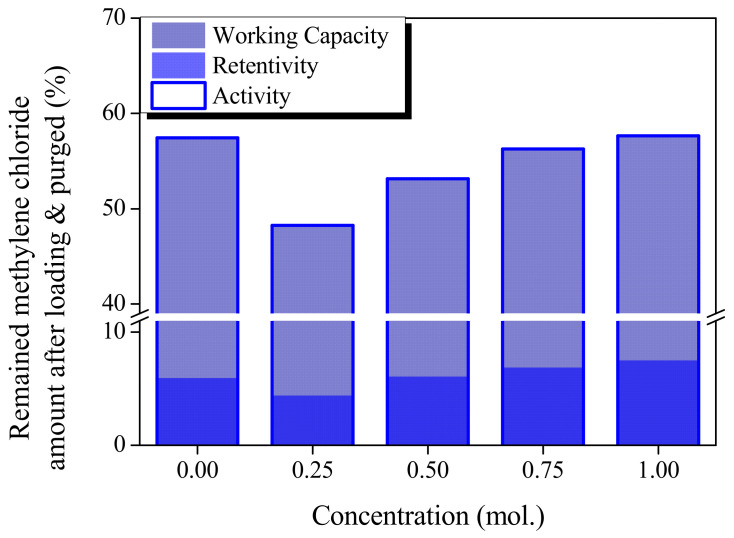
Methylene chloride adsorption–desorption behaviors of cellulose-based activated carbon fibers pretreated with various concentrations of ammonium phosphate.

Methylene chloride working capacity (MWC) is a useful analytical parameter for evaluating MC adsorption/desorption. The MWC, methylene chloride activity (MA), and methylene chloride retentivity (MR) of CACF and AP-CACF were analyzed (Figure 5). MA and MR indicate the amount of MC adsorbed onto the ACF in a saturated state and the residual amount after degassing, respectively. The MWC is defined as the difference between the amount adsorbed at saturation and the amount remaining after degassing. The MR of AP-CACF increased as the concentration of ammonium phosphate increased and ranged from 4.41% to 7.49%. The MR of AP-CACF increased with decreasing mesopore volume (0.06–0.03 cm^3^/g) and mesopore ratio (6.18–3.58%). Generally, ACFs with developed mesopores are known to easily desorb adsorbates during purging [34]. Because the mesopore fraction of CACF (8.33%) is larger than that of AP-CACF (6.18–3.58%), its MR should be low. However, the MR of CACF was 5.94%. Figure 3 confirms that the CACF has hysteresis, which hinders the desorption of the MC. The MA of AP-CACF increased from 48.26 to 57.64% with increasing ammonium phosphate concentration. Generally, MC adsorption occurs mainly in micropores, and the adsorbed MC decreases as the micropore volume decreases. Moreover, the MA of AP100-CACF (57.64%) was closely similar to that of CACF (57.45%). Although the specific surface area and micropore volume of CACF were lower than those of AP-CACF, they adsorbed similar amounts of MC. This is attributed to the different pore diameter distributions of CACF and AP-CACF.

**Figure 6:**
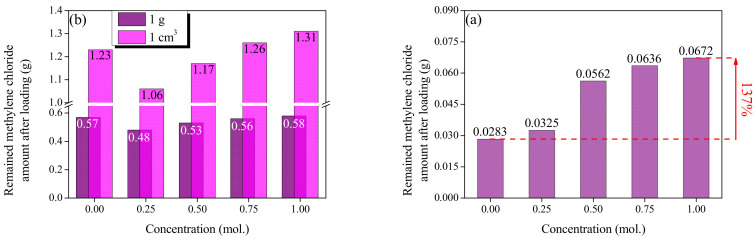
Methylene chloride activity of cellulose-based activated carbon fibers pretreated with various concentrations of ammonium phosphate: (**a**) mass and volume of AP-CACF, (**b**) AP-CACF prepared with the same amount of precursor.

Figure 6 shows the adsorption of MC based on the mass, volume, and total yield of CACF and AP-CACF. Figure 6a exhibits the amount according to unit mass and volume. CACF (1 g) and AP-CACF (1 g) adsorbs 0.57 g and 0.48–0.58 g of MC, respectively. Thus, the AP-CACF adsorbed less or similar amounts of MC compared to the CACF. However, CACF (1 cm^3^) adsorbs 1.23 g of MC, whereas AP100-CACF (1 cm^3^) adsorbs 1.31 g of MC. This corresponds to approximately a 6.5% increase. As shown in Figure 4, CACF and AP-CACF exhibited an increase in density from 2.1458 to 2.2802 g/cm^3^ as the ammonium phosphate concentration increased. Figure 6b shows the MC adsorption amounts of the CACF and AP-CACF prepared with 1 g of cellulose. CACF and AP-CACF adsorbed 0.0283 g and 0.0325–0.672 g of MC, respectively as the ammonium phosphate concentration increased, with a maximum increase of 137%. This suggests that a larger amount of MC can be adsorbed with the same amount of cellulose precursor.

**Figure 7:**
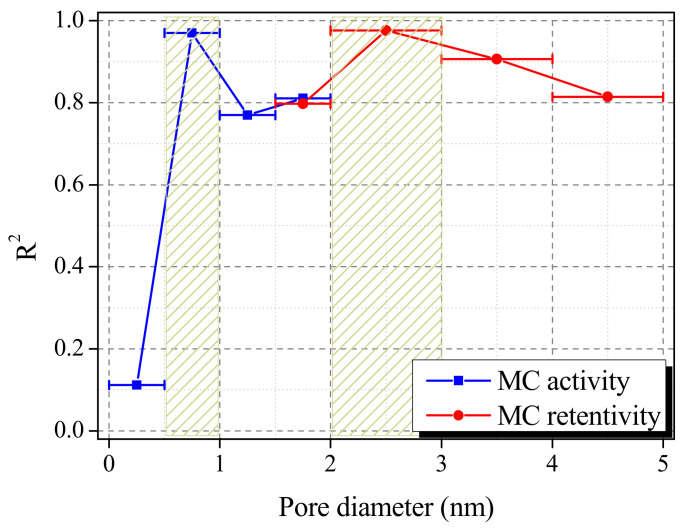
Correlation between methylene chloride activity/retentivity and pore diameter of cellulose-based activated carbon fibers pretreated with ammonium phosphate.

Figure 7 exhibits the correlation between the AP-CACF pore diameter, MA, and MR. This was obtained from plotting the coefficient of determination with MA and MR after plotting the pore volume according to the pore diameter using the nonlocal density functional theory (NLDFT) method. Pore volumes were within a 0.5 nm range for pores below 2 nm and a 1 nm range for pores equal to or larger than 2 nm. Generally, adsorption mainly occurs in micropores, whereas degassing occurs in mesopores [35]. Therefore, MC adsorption showed a correlation with MA and micropores (<2 nm), and MC desorption showed a correlation with MR and mesopores (>2 nm). As exhibited in Figure 7, the MA of AP-CACF depended on pores with a diameter of 0.5–1.0 nm, and the MR depended on pores with a diameter of 2.0–3.0 nm. This implies that micropores (0.5–1.0 nm) and mesopores (2.0–3.0 nm) are dominant in MA and MR, respectively. As shown in Figure 5, the MA of CACF and AP-CACF were similar at 57.45% and 57.64%, respectively. Thus, CACF had several pores with a dominant diameter (0.5–1.0 nm) in MA, and although the micropore volume is lower than that in AP100-CACF, MA is suggested to be similar.

## 3. Experiments

### 3.1. Materials and Preparation

The cellulose-based activated carbon fiber (CACF) was prepared using a cellulose-knitted fabric (Bio-based fiber, Dissol, Jeonju, Republic of Korea) with a fineness of 5 denier. The cellulose-knitted fabrics were treated with ammonium phosphate (ammonium phosphate 98.5%, Samchum Chemical, Seoul, Republic of Korea) at 150 °C for 24 h. The concentrations of ammonium phosphate used for the pretreatment were 0.25, 0.50, 0.75, and 1.00 mol/L. The treated cellulose fabric was washed 2–3 times with distilled water at 80 °C until the pH reached 7 to completely remove ammonium phosphate remaining on the surface. The washed knitted fabric was dried in a vacuum oven at 80 °C for at least 24 h to completely remove the residual water. The CACFs were prepared through stabilization, carbonization, and activation processes. The stabilization process was performed in a tubular heating furnace made of alumina. The inner diameter and length of the heat-treatment furnace were 70 mm and 1200 mm, respectively, with a heating section with a length of 200 mm in the middle of the tubular heating furnace. The cellulose fabric was placed in an alumina crucible and heated. The heating furnace was heated to 300 °C at a heating rate of 5 °C/min and maintained at 300 °C for 2 h. Subsequently, it was naturally cooled to room temperature (25 °C), and an inert atmosphere (N_2_, 200 cc/min) was maintained inside the heating furnace during all the processes.

The stabilized cellulose fabric was carbonized in a tubular heating furnace (alumina). The heat-treatment furnace had a heating section (100 mm) with an inner diameter and length of 80 mm and 1000 mm, respectively. The stabilized cellulose-knitted fabric was placed in the center of the heating section and heated to 800 °C at a heating rate of 5 °C/min for 1 h. Thereafter, the furnace was cooled naturally to room temperature, and nitrogen was supplied at a flow rate of 200 cc/min to maintain an inert atmosphere inside the furnace.

The activation process was performed in a tubular heating furnace made of stainless steel (SAE 310S). The inner diameter and length of the heat-treatment furnace were 70 mm and 1200 mm, respectively, with a heating section with a length of 200 mm in the middle of the tubular heating furnace. The carbonized cellulose fabric was placed in an alumina crucible and heated. The heating furnace was heated up to 900 °C at a heating rate of 10 °C/min, and nitrogen was introduced at a flow rate of 200 cc/min for an inert atmosphere up to the target temperature (900 °C). Next, it was physically activated for 1 h in a steam atmosphere at the target temperature (900 °C). Steam was produced by heating distilled water to 170 °C, and distilled water was supplied at a rate of 0.5 cc/min. After activation, the mixture was cooled naturally to room temperature in an inert atmosphere (N_2_, 200 cc/min).

### 3.2. Property Analysis

The surface morphology of activated carbon fibers pretreated with ammonium phosphate (AP-CACFs) was analyzed using a scanning electron microscope (SEM, AIS 2000 C, Seron Tech Inc., Uiwang, Republic of Korea). The AP-CACF was fixed to a holder (sample size, 1 × 1 cm^2^), and the sample was coated with platinum nanoparticles for 240 s (3 mA) to prevent electron charging before measurement. Images were acquired in a chamber using electrons accelerated to 20 kV at 10^−5^ PA.

The crystal structure of AP-CACF was analyzed using X-ray diffraction (XRD, EMPYREAN, Malvern Panalytical Ltd., Malvern, UK). The analysis used a CuKα radiation source (1.542 λ) and measured 2θ (10–65°) at a rate of 2 °/min. All samples were measured in a knitted form to avoid damaging the crystal structure. The measured patterns were used to calculate the crystallite size using the Scherrer equation.

The textural properties of AP-CACF were analyzed using N_2_/77 K adsorption/desorption isotherms (BET, BELSORP-Max, BEL Japan, Tokyo, Japan), and the specific surface area and total pore volume were calculated using the Brunauer–Emmett–Teller (BET) equation. The specific surface area and total pore volume were calculated based on the adsorbed amount at relative pressures (P/P0) ranging from 0.02 to 0.2. In addition, the micropore volume was obtained from the intercept of the t-plot. Before measurement, the sample was pretreated at 573 K under a pressure < 10^−3^ bar for at least 12 h to remove the residual water.

Total yield was measured using a precision balance (ML304T, Mettler Toledo Ltd., Columbus, OH, USA). The masses of the cellulose precursor and AP-CACF were measured and expressed as a ratio. The densities of CACF and AP-CACF were measured using an automatic pycnometer (Accupyc 1345, Micromeritics, Norcross, GA, USA). Before measurement, the CACF and AP-CACF were dried in a drying oven at 100 °C for 24 h to ensure the complete removal of moisture. CACF and AP-CACF were placed in a 10cc dedicated holder, and their densities were measured. Helium gas was used for the measurement, and the average value was calculated after conducting five measurements.

The adsorption of methylene chloride (MC) was evaluated by placing AP-CACF (1 g) into a U-shaped cell. For adsorption, after placing the cell in a constant-temperature water bath maintained at 25 °C, MC (20,000 ppm) was supplied to the cell at a flow rate of 300 cc/min. The AP-CACF maximally adsorbed MC until there was no mass change. Subsequently, for desorption, N_2_ was supplied for 40 min at a flow rate of 500 cc/min. Methylene chloride working capacity (MWC), methylene chloride activity (MA), and methylene chloride retentivity (MR) were calculated using Equations (1)–(3). The cell weight (*A*), sample and cell weight (*B*), saturated sample and cell weight (*C*), and degassed sample and cell weight (*D*) were measured using a precision balance (ML304T, Mettler Toledo Ltd., Columbus, OH, USA) [36].

(1) $$MWC=\frac{C-D}{B-A}\times 100$$

(2) $$MA=\frac{C-B}{B-A}\times 100$$

(3) $$MR=\frac{D-B}{B-A}\times 100$$

## 4. Conclusions

In this study, cellulose-based activated carbon fibers (CACFs) were prepared by pretreating cellulose fibers with different concentrations of ammonium phosphate (0.00, 0.25, 0.50, 0.75, and 1.00 mol/L). From the results, the specific surface area and total pore volume of the cellulose-based activated carbon fiber pretreated with ammonium phosphate (AP-CACF) were 2060–1920 m^2^/g and 1.02–0.83 cm^3^/g, respectively. In addition, the total yield of AP-CACF was 6.78–11.59%, which was improved compared to that of CACF (4.97%). The increase in AP-CACF yield is closely related to the crosslinking density of cellulose. As the crosslinking density increases, the yield can be increased by preventing the excessive oxidation of cellulose molecules during heat treatment. The correlation between textural properties and the adsorption/desorption of MC has significant implications for developing adsorbents for removing MC. The micropores in the 0.5–1.0 nm diameter range mainly control MC adsorption, while the mesopores in the 2.0–3.0 nm diameter range mainly control MC desorption.

Owing to the rapid growth of the battery industry, methylene chloride emissions are also increasing rapidly. Therefore, developing efficient adsorbents for MC is necessary. In this study, AP-CACF with excellent MC adsorption and improved yield was prepared. The knitted AP-CACF is highly applicable in the industry as an adsorbent for MC removal. This also suggests that the efficiency and cost of the adsorbent for MC removal can be reduced, and that it has high potential for use in the industry.

## Figures and Tables

**Table 1:** Textural properties of cellulose-based activated carbon fibers pretreated with various ammonium phosphate concentrations (0.25–1.00 mol).

Sample	S_BET_^a^ (m^2^/g)	V_Total_^b^ (cm^3^/g)	V_Micro_^c^ (cm^3^/g)	V_Meso_^d^ (cm^3^/g)	F_Micro_^e^ (%)	F_Meso_^f^ (%)	Act.Yield ^g^ (%)
CACF	1570	0.72	0.66	0.06	91.67	8.33	25.65
AP025-CACF	2060	1.02	0.96	0.06	93.82	6.18	19.12
AP050-CACF	2030	0.92	0.88	0.04	96.03	3.97	29.22
AP075-CACF	2000	0.88	0.84	0.03	96.23	3.77	35.95
AP100-CACF	1920	0.83	0.80	0.03	96.42	3.58	37.98

^a^ S_BET_: specific surface area (Brunauer–Emmett–Teller method), ^b^ V_Total_: total pore volume (BET method), ^c^ V_Micro_: micropore volume (t-plot method), ^d^ V_Meso_: mesopore volume (V_Total_ − V_Micro_), ^e^ F_micro_: micropore fraction, ^f^ F_meso_: mesopore fraction, ^g^ Yied: $\frac{weight\;of\;activated\;sample}{weight\;of\;carbonized\;sample\;input}\times 100$.

## Data Availability

Not applicable.
